# Practical templates for digital health ethics applications in Sweden: lessons from a sensor-based monitoring study

**DOI:** 10.3389/fdgth.2026.1745662

**Published:** 2026-03-27

**Authors:** Samaneh Zolfaghari, Rose-Marie Johansson-Pajala, Lena Marmstål Hammar, Masoud Daneshtalab

**Affiliations:** 1 School of Innovation, Design and Engineering, Mälardalen University, Västerås, Sweden; 2 School of Health, Care and Social Welfare, Mälardalen University, Västerås, Sweden

**Keywords:** digital health, ethics application, older adults, research ethics, sensor-based monitoring, Sweden, templates

## Abstract

Obtaining ethical approval for digital health research involving vulnerable populations presents significant challenges for researchers, particularly when navigating complex regulatory frameworks like Sweden’s ethical review system. Despite official guidelines, researchers often struggle to translate general principles into concrete application documents that satisfy review authorities. This paper presents practical, reusable templates developed through the successful preparation of an ethics application for a radar-based monitoring study of older adults in Sweden. The application, involving cross-border data handling and vulnerable populations, received first-submission approval from the Swedish Ethical Review Authority (Etikprövningsmyndigheten) without reviewer comments. We provide structured templates for key application sections, identify common pitfalls, and offer evidence-based guidance for researchers preparing similar applications. These resources are practice–grounded (one approved applications plus practice-based reflections) and are intended to reduce administrative burden and improve application quality; they should be adapted to local institutional requirements.

## 1 Introduction

The rapid advancement of digital health technologies, particularly sensor-based monitoring systems for older adults—offers unprecedented opportunities to promote independent living and enable early detection of health deterioration (1). These innovations pave the way for timely interventions that can improve quality of life and empower caregivers to provide more effective support (2, 3).

However, the deployment of such technologies also introduces complex ethical challenges concerning privacy, informed consent, data governance, and the protection of vulnerable populations (4, 5). Addressing these issues requires careful consideration throughout the research process, from study design to data management and participant communication.

In Sweden, all research involving human subjects must, according to the Ethical Review Act (SFS 2003:460), obtain approval from the Swedish Ethics Review Authority (Etikprövningsmyndigheten).^1^ The Ethics Review Authority includes lay representatives (5) in addition to members with scientific expertise (10). They evaluate applications in accordance with the Ethical Review Act, the EU General Data Protection Regulation (GDPR) (6), and emerging frameworks such as the EU AI Act (7). While legal frameworks establish formal boundaries for research conduct, ethical responsibility extends beyond regulatory compliance. The focus is that when human participants are involved in or affected by research, ethical reflection is grounded in four core principles: beneficence, non-maleficence, respect for autonomy, and justice (8). These principles are historically rooted in foundational documents such as the Nuremberg Code and the Declaration of Helsinki, which continue to inform contemporary research governance, including emerging digital contexts. Ethical considerations are therefore as integral to study planning as any other methodological component.

Regulations provide structural requirements; however, researchers carry a fundamental responsibility to ensure that research is conducted ethically, transparently, and reliably across all stages—from study design and data collection to the management, reporting, and dissemination of findings (9). This underscores the necessity for robust ethical competence within the organizations conducting the research and this needs to be explicit in the ethical application for the planned research.

Due to the rapid growth of technology research in health care, many researchers, especially those from technical disciplines, often lack experience in ethical application as it historically has not been needed. Hence, concrete, field-tested guidance for preparing Swedish ethics applications is needed. Swedish ethical standards must also be upheld internationally, meaning that researchers cannot relocate studies to countries with weaker ethical regulations. As a consequence, technology researchers working with, for example, sensors, AI tools, predictive modeling, or human–computer interaction may need ethical approval in Sweden even when similar projects conducted abroad do not. Historically, technical research has not required ethical review, yet new technologies continuously reshape ethical challenges. Sweden therefore expects researchers to demonstrate a clear sense of personal ethical responsibility in their applications, and this responsibility must be upheld throughout the project in accordance with the Ethical Review Act. For researchers without prior training in ethics, writing such applications can be particularly challenging.

Ethical practice includes that the researchers demonstrate respect for their participants, for themselves and in a larger perspective also for society. This includes embedding empathy, reflexivity, and care which needs to be explicit when conducting the research and thus explained in the sections of the application.

Despite the availability of official guidelines, many researchers, especially those from technical disciplines or involved in international collaborations, often lack concrete, field-tested guidance for structuring Swedish ethics applications. Poorly prepared submissions often lead to extended review timelines (10), requests for clarification, or resubmission (11). These delays can disrupt project schedules, affect funding cycles, and limit research quality when ethical aspects are handled reactively rather than proactively. Moreover, current resources tend to emphasize legal compliance rather than practical translation of ethical principles into well-organized, reviewer-ready submissions. This gap between regulatory expectations and researcher practice highlights the need for reusable applied templates that align with the requirements of the Swedish Ethical Review Authority while supporting efficiency, transparency, and ethical rigor in digital health research.

To address these challenges, this article presents structured, practical, evidence-based templates derived from a successful ethics application for a radar-based monitoring study of older adults that were reviewed by the Swedish Ethical Review Authority. It does not present empirical findings derived from a representative sample of ethics applications. The ethics application for this study was approved on the first submission without the reviewer’s comments or required modifications, indicating that its structure, clarity, and ethical reasoning were closely aligned with the expectations of the Swedish Ethics Review Authority.

The paper provides a justification for ethical consideration in technological research. Ethical considerations are crucial in this field because emerging digital tools can generate risks during data collection and later when research results or technologies are applied in new contexts beyond the control of researchers. Moreover, the rapid development and dissemination of new technologies often outpace ethical deliberation, increasing the risk that important ethical questions are overlooked (9). Sweden is to be seen as a suitable case for highlighting ethical applications in digital health research, as it follows a rigorous process governed by national legislation and since research ethics in most countries are grounded in similar fundamental principles, the topic should also be of interest to an international audience. Sweden therefore functions as an illustrative example rather than the conceptual focus of the work. Its centralized Ethical Review Authority has legally binding decisions and includes both scientific and lay members. The country’s highly digitalized health system, widespread use of personal identity numbers, and strong alignment with GDPR create a setting where digital monitoring technologies interact with a transparent and well-regulated ethical review process. Sweden is not unique, but it clearly shows how technological innovation must align with rigorous ethical governance.

To support the development of the templates and situate the work within existing scholarship, we conducted a focused narrative review of literature on research ethics committees, digital-health governance, and cross-border data management. Between January 2024 and October 2025, we searched Scopus, Web of Science, and PubMed using terms such as “research ethics committee/ethical review,” “digital health,” “sensor-based monitoring,” “AI in healthcare,” and “ethics application templates.” We supplemented this with targeted searches of national ethics-authority websites, EU policy documents, and institutional compliance resources, and we screened reference lists of key publications. All literature identification, full-text retrieval, critical evaluation, and synthesis were conducted exclusively by the authors. Findings from this review directly informed the structure and emphasis of the templates, particularly the need for plain-language explanations, clear risk–benefit reasoning, precise data-handling terminology, and transparent documentation of international data flows.

Therefore, the purpose of this study is to extract transferable lessons and articulate ethical reasoning processes that can assist researchers in similar contexts. The templates presented in this study should therefore be understood as illustrative scaffolds intended to support ethical reflexivity and clarity, rather than as universally generalizable models.

The remainder of this paper is organized as follows:

- Section 2 reviews existing resources and identifies the need for practical templates. It outlines the case study which is an international radar-based monitoring project, describes the development of templates and the iterative process used.
- Section 3 presents practical templates for ethics applications, offering guidance and examples tailored to both context-specific and more complex sections.
- Section 4 summarizes best practices, practical insights, and recommendations.
- Section 5 presents the main limitations of this work.
- Section 6 highlights main contributions and outlines future work.

## 2 Assessment of guideline options and implications

### 2.1 Review of existing guidelines

Although there are several resources for ethics applications in Sweden and the EU, most provide general principles rather than practical templates. The Swedish Ethical Review Authority offers official guidelines (12), but these focus on regulatory requirements rather than application structure. Institutional guidance from universities such as Karolinska Institute (KI) (13) provides additional detail, but remains general. While regulations such as the GDPR (6) and the EU AI Act (7) establish legal boundaries, they do not provide detailed guidance on how ethical reasoning should be operationalized in specific research contexts. This interpretive work must be undertaken by researchers, ethics committees, and scholarly communities.

Previous studies have also reported persistent challenges in the ethics review process, including unclear or overly technical language (14), insufficient detail on data handling and consent procedures (11), and inconsistent understanding of risk–benefit assessment (15). Broader analyses also highlight the uncertainty of researchers in translating high-level ethical frameworks, such as GDPR, into concrete, compliant protocols (4, 5). It is also noteworthy that since the Ethics Review Authority includes lay representatives, it underscores the need for plain, accessible language, in Swedish, to ensure that all members can adequately assess submissions. Table 1 summarized practical issues that are consistent with our findings in the literature. We have also presented actual problems, and proposed solutions for these issues.

**Table 1:** Common pitfalls and solutions.

Issue	Problem	Solution
Technical language	Reviewers can’t evaluate what they don’t understand	Provide plain-language explanations first, technical details second
Vague data management	“Secure storage” without specifics	Specify services, locations, and security measures
Anonymization confusion	Misusing terms	Use “pseudonymized” if codes could theoretically be re-identified
Missing risk-benefit	Focusing only on benefits	Explicitly address potential risks and mitigation measures
Consent gaps	Unclear who consents how	Provide step-by-step consent procedures with timelines and responsible parties
International transfer	Not addressing GDPR	Specify the legal mechanism
Incomplete timetable	Optimistic or vague timelines	Include dependencies, realistic durations, and buffer time

These barriers are particularly pronounced in multidisciplinary areas such as digital health, where technical complexity intersects with human subject research.

Page and Nyeboer (11) proposed a model of the ethics review process that emphasizes the roles of key stakeholders, including researchers, members of the ethics board, institutional administrators, and support staff. Their study identified inefficiencies related to incomplete submissions and lack of researcher familiarity with ethical standards, suggesting that improved guidance and educational tools could reduce processing delays. Turner (16) further argued that researchers should demonstrate foundational knowledge of legal and ethical standards before submitting applications, to enhance both compliance and review efficiency. Such perspectives underscore the need for practical, accessible resources that support researchers in preparing ethically robust applications from the outset. Research ethics review systems face critiques for bureaucracy, inconsistent interpretations, and rigid procedures that can slow or limit valuable research. Scholars highlight tensions between formal compliance and meaningful ethical reflection, especially in interdisciplinary or technology-driven work. Recognizing these concerns is important: greater transparency in application structures and ethical reasoning could improve both efficiency and the quality of ethical deliberation.

Complementary analyses in the social sciences have highlighted a mismatch between the biomedical orientation of most ethics review systems and the needs of applied or participatory research (15). Overly restrictive or risk-averse interpretations of ethics regulations may inadvertently hinder research that is socially valuable or time-sensitive. Macnamara (15) further noted that the complexity and length of application forms themselves can act as procedural barriers, particularly in interdisciplinary projects that involve behavioral, environmental, or digital monitoring components.

This paper addresses these gaps by presenting structured templates for key sections of Swedish ethics applications, specifically tailored for digital health research involving sensor-based monitoring and international data collection. The recommendations are derived from a successfully approved first submission, demonstrating strong alignment with the Authority’s expectations. To our knowledge, no existing resource provides reusable, field-tested templates based on successful applications. These materials are particularly useful for researchers, research coordinators, and ethics advisors at Swedish institutions, as well as international collaborators partnering with Swedish teams. Ethics committee members may also find the templates valuable as examples of clear and well-structured applications. The structure proposed here can be reused and adapted for similar projects.

The templates are especially suited for projects involving non-invasive sensor-based monitoring, such as ambient, radar-based technologies, and for studies focusing on older adults or other vulnerable populations. They also apply to projects involving cross-border data collection and AI-driven health monitoring systems. Furthermore, the templates integrate privacy-preserving design principles that support both ethical integrity and innovation. Although optimized for the Swedish research ethics framework, many principles extend to other EU contexts. The focus is primarily on observational monitoring studies rather than interventional clinical trials, assuming a basic understanding of GDPR and fundamental research ethics principles.

### 2.2 Context: sensor-based indoor human behavior monitoring

To illustrate the practical application of the templates, we have considered the *Secure Detection of Wandering Behavior (SDWB)* project.^2^ This project is an international collaboration between Mälardalen University (MDU) (Sweden), the University of Waterloo (Canada), and Gold Sentintel Inc. (Canada). The aim is to develop privacy-preserving AI models for detecting wandering behavior in older adults using frequency-modulated continuous-wave (FMCW) radar technology. The project emphasizes contactless, non-invasive monitoring without cameras or microphones, secure cross-border data management between Canada and Sweden, and the protection of participant privacy through pseudonymization.

The SDWB project presented several ethical complexities that shaped the development of our templates. Key challenges included obtaining valid informed consent from participants with cognitive impairments, ensuring compliance with both GDPR (EU/Sweden) and PIPEDA (Canada) in cross-border data governance, and effectively communicating the principles of radar-based sensing to non-technical ethics reviewers. The project also required balancing privacy and surveillance considerations to promote safety while respecting participants’ autonomy, establishing secure international data transfer protocols, and maintaining transparency in the design and explanation of AI and deep learning methods.

The ethics application was submitted to the Swedish Ethics Review Authority in April 2025 and approved on first submission in May 2025 (dnr: 2025-02828-01), without requests for revision or clarification. This positive result indicates that the structure, language, and supporting documentation of the application effectively addressed the Authority’s evaluation criteria. We attribute this success to several key factors:

- Early interdisciplinary collaboration between computer scientists and healthcare researchers.
- A structured, template-based approach for each section of the application.
- Clear, plain-language explanations alongside technical details.
- Comprehensive data management documentation and protocols.
- Institutional support letters confirming governance and oversight structures.
- Emphasis on participant benefit and minimal risk.

These successful practices have been distilled into the templates presented in Section 3, providing guidance for future ethics applications in similar contexts.

### 2.3 Methodology: development of templates

The practical templates and recommendations presented here are derived from one successfully approved Swedish ethic application including our own submission for a radar-based monitoring study and supplemented by structured, practice-based reflections and anecdotal observations gathered during interdisciplinary preparation. Accordingly, the “data” supporting our templates consist of document artifacts (completed application sections, attachments) and process insights (e.g., recurring reviewer expectations, terminology that improved clarity), not statistical outcomes from a larger sample of projects.

The initial drafting phase involved structuring the responses according to the official Swedish Ethical Review Authority application form and incorporating KI institutional ethical or research governance framework. In the subsequent interdisciplinary review phase, healthcare researchers refined clinical relevance and patient-centered language, while data protection officers ensured compliance with GDPR and PIPEDA. International collaborators contributed feedback on cross-border data governance and privacy-preserving practices. Following approval, the material was refined to ensure terminological consistency, simplify technical explanations, and strengthen data management documentation. The resulting templates distill these lessons into adaptable structures intended to support future applicants in preparing well-aligned and ethically sound submissions.

**Figure 1:**
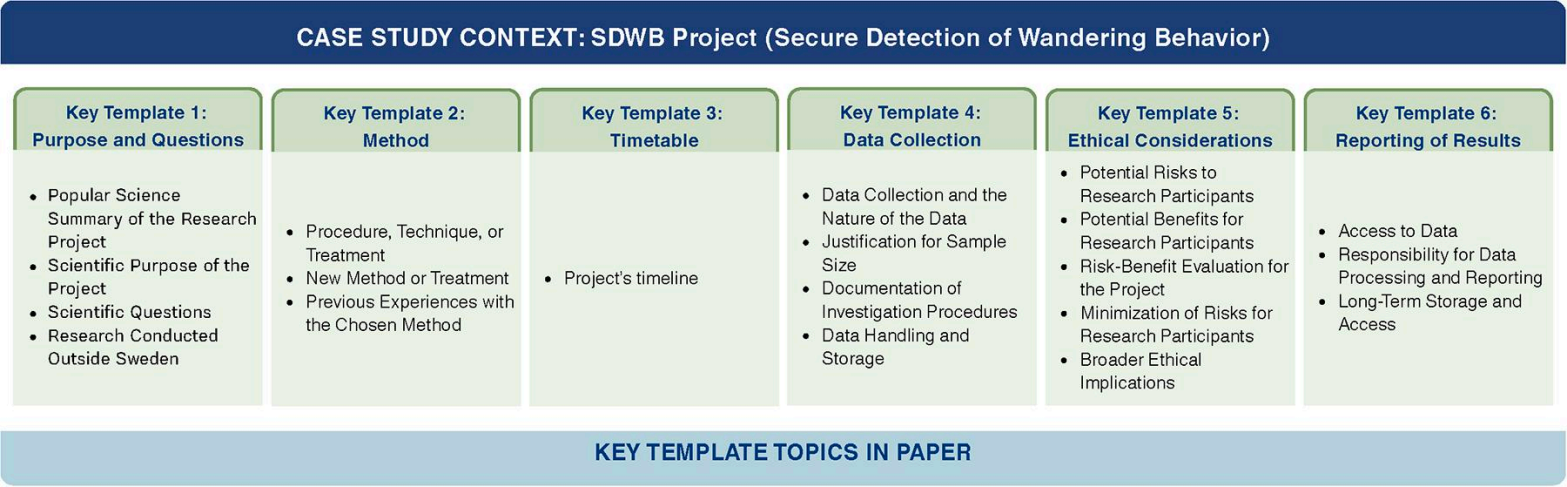
An overview of key practical template topics in this paper.

Each template is designed to help researchers structure their responses according to the requirements of the Swedish Ethical Review Authority. For each template, there is a clear **purpose statement** describing what the authority aims to evaluate, a **structural guidance** outlining how to organize the response effectively, and **examples** illustrating how to address the questions in practice. In Figure 1 the practical templates considered in this study are demonstrated.

## 3 Practical templates and actionable recommendations

This section provides actionable templates (as illustrative structural elements), and recommendations distilled from a successfully approved application. The Swedish Ethical Review Authority’s application portal comprises 13 sections, each containing multiple questions that must be completed carefully before submission. Table 2 summarizes all sections of the application and marked those addressed in this paper. Several sections of the application are relatively straightforward and can be completed using the official guidance provided by the Authority (i.e., *Main tasks*, *Type of research—initial questions*, *Research participants*, *Information and consent*, *Register information*, and *Economic conditions*), while others are more complex and typically require additional support. The selected sections represent the aspects most relevant to research projects involving cross-border collaboration, particularly when data from human research participants are collected outside Sweden.

**Table 2:** Checklist of application sections covered in this paper.

Application section	Included in paper
Main tasks	□
Type of research—initial questions	□
Purpose and questions	✓
Method	✓
Timetable	✓
Data collection	✓
Ethical considerations	✓
Research participants	□
Information and consent	□
Register information	□
Results from animal experiments	□
Reporting of results	✓
Economic conditions	□

In addition to these 13 sections, the application includes two supplementary sections: *Attachments* and *Signatures*. The *Attachments* section typically includes:

- **Research Plan**—A detailed description of the study, written for an expert audience. It should follow the specified guidelines and include the scientific question, background and area overview, project description, significance, and any preliminary results. This section of the ethics application may be written in either Swedish or English.
- **Information for Research Subjects and Consent Forms**—All materials provided to participants, guardians, or relatives (both written and oral) must be attached. The Authority recommends using the official support template available at www.etikprovningsmyndigheten.se. These documents must be submitted in Swedish.
- **CV of the Responsible Researcher**—The CV of the primary researcher must be attached. In exceptional cases, a non-doctoral researcher may be accepted if supervised by a doctoral researcher, whose CV and a certified supervision statement are also included. CVs may be submitted in Swedish or English.

These attachments allow the Authority to assess the scientific rationale, participant protections, and qualifications of the research team. The *Signatures* section requires signatures from both the authorized representative and the responsible researcher, confirming accountability and credibility of submitted application. Table 3 summarizes the key sections addressed in the following subsections. While these sections are standard requirements in Swedish ethics applications, they pose particular challenges in the context of this study, forming the basis for the templates and guidance that follow.

**Table 3:** Key sections in Swedish ethics applications.

Sections	Key templates	Purpose	Critical elements
3.1	Purpose and questions	Explain research in accessible language and define research aims and rationale	Public-facing summary, societal relevance, clear objectives, hypotheses, measurable outcomes, and jargon-free
3.2	Method	Describe what will be done	Procedures, technologies, data collection protocols, ethical compliance
3.3	Timetable	Project schedule and dependencies	Start/end dates, phase-wise activities, dependencies, deliverables
3.4	Data collection	How data is gathered and stored	Sample size justification, collection procedures, storage security, GDPR compliance, pseudonymization
3.5	Ethical considerations	Analyze risk-benefit balance	Potential risks, benefits, mitigation measures, broader ethical implications
3.6	Reporting of results	How data is analyzed, reported, and stored long-term	Access restrictions, responsible personnel, dissemination, secure storage, retention, future access

### 3.1 Key template 1: purpose and questions

#### 3.1.1 Popular science summary of the research project

**Purpose:** This section is critical for reviewers who may not be technical experts, shaping their first impression. Support your arguments with relevant literature and include citations where appropriate to enhance the credibility of your work.

**Structure:**

- *Introduction/background*: Describe the societal problem or research need.
- *Target population*: Specify the affected group(s).
- *Research approach*: Summarize methods or technology.
- *Expected impact*: Describe benefits and importance.
- *Collaboration & scope*: Include partners, locations, and scale.

**Example (adapted from SDWB):**

The project aims to improve remote monitoring of older adults’ activities and movements using advanced, privacy-preserving technology. With the aging population, the proportion of older people is increasing rapidly. In the EU, the elderly dependency ratio is expected to rise from 25.4% in 2008 to 53.5% in 2060 (3). In Sweden, 20% of the 10.5 million inhabitants are currently over 65, projected to reach 23% by 2040 (17). Aging is often accompanied by cognitive decline and mobility issues, creating societal challenges and increasing demand for caregivers (1). People with dementia, in particular, may exhibit wandering behavior—moving aimlessly or repetitively—which poses safety risks such as falls (18).

Existing detection methods rely on infrequent clinical assessments or intrusive technologies such as cameras or wearables, limiting real-world applicability. This project uses non-invasive radar-based sensors to continuously monitor movement patterns and activities in long-term care settings. Analyzing these sensor streams enables detection of early signs of cognitive or physiological decline and wandering behavior (1), supporting timely interventions to improve quality of life and caregiver support.

Non-invasive monitoring is less disruptive, preserves privacy, and respects autonomy, as it does not require wearable devices or cameras. Continuous data collection allows subtle behavioral changes to be detected, facilitating early interventions (2, 3).

The project is a collaboration between Swedish health science and technology departments and Canadian research institutes, including the University of Waterloo. Data collection occurs at Trinity Village, a long-term care facility in Canada. The system aims to increase safety for residents and provide healthcare professionals with tools for early dementia diagnosis.

#### 3.1.2 Scientific purpose of the project

**Purpose:** Explain what the project intends to achieve scientifically.

**Structure:**

- *Overall aim*: One or two sentences describing the main goal.
- *Specific objectives*: Use bullet points for clarity; include measurable outcomes, if relevant.
- *Rationale*: Briefly explain why this purpose is scientifically important.

**Example (adapted from SDWB):**

This project develops a safe, reliable, and privacy-preserving method to detect and analyze dementia-related movement and activity patterns using indoor radar sensors. Data-driven models identify early behavioral indicators such as aimless or repetitive movement (19), enabling proactive care interventions.

**Specific Objectives:**

- Validate sensor accuracy in identifying activity levels and movement patterns.
- Correlate movement patterns with healthcare data, e.g., links between disorientation and cognitive decline.
- Develop analytical models to detect movement trends that signal health issues and enable early intervention.

Combining sensor data with healthcare information aims to improve safety in long-term care, optimize care strategies, and support early detection and intervention.

#### 3.1.3 Scientific questions

**Purpose:** Identify the research questions that guide methodology and analysis.

**Structure:**

- *Key questions:* List 3–5 concise questions, focused questions that align with the project’s scope and timeline. Use bullet points.
- *Scope:* Indicate whether questions concern detection, modeling, ethics, or generalizability.
- *Connection:* Link questions back to the overall aim of the project.

**Example (adapted from SDWB):**

The project addresses the following key scientific questions:

- Detection of wandering behavior: How can non-invasive environmental sensors and data-driven models reliably identify wandering behavior associated with cognitive impairment in indoor environments?
- Feature extraction and behavioral patterns: Which movement features derived from sensor data best distinguish between healthy individuals and those with cognitive impairment?
- Generalizability and adaptability: How can data augmentation and synthetic data improve model performance across different homes and individual movement patterns?
- Privacy-protected monitoring: How can motion monitoring ensure privacy while maintaining accuracy in detecting behavioral anomalies?
- Ethical and practical considerations: What ethical and technical challenges arise from continuous monitoring of people with dementia, and how can these be mitigated in practice?

These questions shape the project’s methodological design and support its overarching goal of developing an ethical, effective, and scalable system for early detection of cognitive decline.

#### 3.1.4 Research conducted outside Sweden

**Purpose:** Clarifies which components of the research are conducted outside Sweden. This enables reviewers to assess compliance and the integrity of international collaborations.

**Structure:**

- *Confirmation*: Yes/No for research outside Sweden.
- *Specific parts outside/inside Sweden*: Describe activities (e.g., recruitment, data collection, analysis).
- *Procedures abroad*:
  - Locations and institutions.
  - Participant groups and recruitment.
  - Data collection and handling.
  - Ethical safeguards (pseudonymization, privacy measures).
  - How data informs the Swedish study.

**Example (adapted from SDWB):**

Research outside Sweden is conducted at Trinity Village, Canada, with Waterloo University. Activities include participant recruitment, experiments, data collection, and management. Participants include healthy volunteers simulating older adults and residents of long-term care facilities using non-invasive sensors. All data are de-identified and pseudonymized before sharing.

Data collected supports baseline datasets to validate monitoring methods before applying similar analyses in Sweden, enabling a comprehensive understanding of movement patterns and early indicators of health decline.

### 3.2 Key template 2: method

#### 3.2.1 Procedure, technique, or treatment

**Purpose:** Describe what will be done locally (in Sweden) regarding methods, procedures, and techniques.

**Structure:**

- *Scope of local research*: Specify activities (data processing, analysis, participant interaction).
- *Data description*: Type of data used (e.g., pseudonymized, de-identified) and source.
- *Procedure/technique overview*: Steps in bullet form, e.g.:
  - Data preprocessing (cleaning, structuring)
  - Feature extraction
  - Analytical modeling or computational methods
  - Validation or reliability assessment
- *Data reliability measures*: Quality control, data augmentation, separate training/testing.
- *Ethical and legal considerations*: Data protection, privacy preservation, regulatory compliance.
- *Other notes*: Limitations or methodological constraints.

**Example (adapted from SDWB):**

Data collection occurs in Canada. In Sweden, the project focuses solely on processing and analyzing pseudonymized movement and activity data. No participant interaction or collection of personal data is performed locally.

Local research activities include:

- Preprocessing—Cleaning and structuring pseudonymized sensor data.
- Feature extraction—Identifying movement patterns indicative of cognitive impairment.
- Data-driven modeling—Classifying activity patterns and detecting anomalies.
- Validation—Statistical evaluation using cross-validation and sensitivity analysis.

To ensure data reliability:

- Data undergoes strict quality control before transfer.
- Data augmentation enhances generalizability.
- Models are trained/tested on separate datasets to avoid overfitting.
- Compliance with Swedish and Canadian regulations is strictly maintained.

Other:

- The data-driven model cannot be easily applied to data input coming from a camera instead as there is too much difference between data from non-invasive environmental sensors and data from the camera. Various researchers around the world have tried to make this conversion but have not succeeded.
- Modifying these data-driven models to analyze movement patterns in a crowd or to be used for military purposes requires a major redesign.

#### 3.2.2 New method or treatment

**Purpose:** Explain novelty compared to standard methods.

**Structure:**

- *Clarify scope*: Confirm no local participants are affected.
- *Novelty*: Describe innovations (e.g., data-driven modeling, analytical techniques).
- *Comparison with standard methods*: Highlight differences from conventional approaches.
- *Data handling innovations*: Include techniques like data augmentation or model generalization.
- *Impact on routine*: Confirm participants’ routines remain unchanged.

**Example (adapted from SDWB):**

No new treatment is applied locally; research is limited to secondary analysis of pseudonymized data.

Novelty lies in applying data-driven models to detect cognitive decline patterns from radar-based environmental sensors, rather than relying on conventional clinical assessments or wearable devices. Data augmentation and model generalization improve applicability across different indoor environments, with no impact on participant routines.

#### 3.2.3 Previous experiences with the chosen method

**Purpose:** Demonstrate familiarity and credibility with the method.

**Structure:**

- *Comparison with related research*: Cite key studies and differences.
- *Team experience*: Expertise of investigators in sensor-based analysis, modeling, and care.
- *Global experience/context*: Similar methods internationally and gaps addressed.

**Example (adapted from SDWB):**

The study “Alzheimers och fallolyckor kan förutspås med hjälp av radar”^3^ is relevant, but focuses on gait stride length. This project examines holistic indoor movement patterns to detect dementia-related behavior.

Experience of the team includes sensor-based monitoring, AI-driven modeling, and geriatric care. Globally, environmental sensors are increasingly used in dementia detection and fall prevention (1, 20, 21). Unlike prior studies, our approach emphasizes privacy-preserving, continuous, real-world monitoring in long-term care settings.

### 3.3 Key template 3: timetable

**Purpose:** Provide a clear overview of the project’s timeline, showing when each major activity will occur and how they interrelate. This helps reviewers assess feasibility and alignment between objectives, methods, and resources.

**Structure:**

- *Start and end dates:* Indicate the expected project start and completion dates. Adjust project timeline; it should start after ethical approval is granted.
- *Phases and duration:* Divide the project into major phases (e.g., data collection, preprocessing, analysis, dissemination) and specify the approximate months or quarters for each.
- *Dependencies:* Briefly describe how phases depend on or build upon one another.
- *Deliverables:* Note key outcomes such as model validation, reports, or publications linked to each phase.

**Example (adapted from SDWB):**

**Expected start date:** 2025/06/01

**Expected end date:** 2027/05/01

**Timetable for research activities in Sweden:**

- Months 1–4: Data Collection and Preparation
  Canadian partners collect movement data via non-invasive sensors. Data are pseudonymized and securely transferred to the Swedish research team.
- Months 5–9: Data Preprocessing
  Clean and organize data to ensure quality; perform exploratory analyses to identify trends and potential artifacts.
- Months 10–15: Model Development
  Extract motion features, apply data augmentation techniques, and develop data-driven models for behavioral detection.
- Months 16–21: Validation and Optimization
  Conduct model evaluation, optimize algorithms, and assess generalizability across datasets.
- Months 22–24: Final Analysis and Dissemination
  Perform final validations, prepare manuscripts, and present results at conferences.

Each phase builds on the previous one to ensure a logical progression from data acquisition to scientific dissemination. Results will be shared during or shortly after the project period.

### 3.4 Key template 4: data collection

#### 3.4.1 Data collection and the nature of the data

**Purpose:** Describe how data will be collected in Sweden (or locally) and the type of data used.

**Structure:**

- *Local data collection:* State whether primary data will be collected in Sweden or if only pre-collected data will be analyzed.
- *Nature of data:* Specify data type, sensor technology, and measurement properties.
- *Collection procedure (if applicable):* Summarize acquisition phases, equipment used, and participant involvement.
- *Privacy considerations:* Confirm the absence of personal identifiers, images, or audio, and outline pseudonymization procedures.

**Example (adapted from SDWB):**

No primary data collection is conducted in Sweden. All data is collected, pseudonymized, and pre-processed by Canadian partners before analysis by the research team at MDU.

Nature of the data: The dataset consists of pseudonymized motion data collected with non-invasive FMCW radar-based sensors in home environments. No personal identifiers or sensitive information are included. Each dataset is labeled as either representing a healthy individual or a person with cognitive impairment, along with basic activity labels. FMCW radar sensors measure motion and distance with high precision by emitting continuous signals and analyzing their reflections. These sensors are non-invasive, privacy-preserving, and reliable under varying lighting conditions—ideal for home and care environments.

Data collection in Canada: Data collection occurs in two phases:

1. Controlled activity recording: Healthy volunteers simulate daily activities (e.g., sitting, standing, walking) in empty rooms at Trinity Village. Radar systems record movements to create labeled reference data for training and validating algorithms.
2. Resident monitoring: Radar devices installed in residents’ rooms continuously capture movement data such as time spent sitting, getting out of bed, or restlessness. Data is pseudonymized and correlated with de-identified health records to identify potential health trends.

Only motion-based radar signals are collected—no images, audio, or personal identifiers. Data is pseudonymized following approved ethical protocols, ensuring that only activity and movement information is shared.

#### 3.4.2 Justification for sample size

**Purpose:** Explain the reasoning behind dataset size and number of participants.

**Structure:**

- *Controlled data:* Describe participant count and rationale for simulations used in algorithm training.
- *Real-world data:* Indicate the number of monitored participants, expected duration, and statistical justification.
- *Data volume:* Provide an estimate of total instances or observations and justify adequacy for analysis.

**Example (adapted from SDWB):**

The study ensures that collected data is sufficient for meaningful statistical analysis and reliable conclusions regarding movement patterns and health indicators.

A minimum of 10 healthy adults will participate in controlled activity simulations at Trinity Village to provide diverse movement patterns under standardized conditions. These labeled recordings serve as training and validation data for algorithm development.

Long-term radar-based monitoring will involve at least 20 residents in care apartments. This sample size, based on prior studies in activity recognition, offers adequate statistical power to detect behavioral trends related to cognitive or physical decline. Data will be continuously collected over several months to capture both short-term variations and long-term patterns.

To establish statistically significant correlations between movement behavior and health status, the dataset will comprise several hundred thousand motion instances. Combining controlled and real-world data enhances model generalization across environments and individuals, supporting robust validation of radar-based monitoring methods in care settings.

#### 3.4.3 Documentation of investigation procedures

**Purpose:** Describe how procedures and data handling will be logged and documented.

**Structure:**

- Automated logging with time-stamping and secure storage.
- Facility permissions and activity labeling for accuracy.
- Support for validation and reproducibility.

**Example (adapted from SDWB):**

All data is automatically logged, time-stamped, and securely stored on encrypted servers. Logging is performed with facility permissions to enable accurate labeling of activities, supporting reliable classification and model validation.

#### 3.4.4 Data handling and storage

**Purpose:** Explain how data will be securely stored, accessed, and used in Sweden.

**Structure:**

- **Data transfer:** Specify transfer method and encryption measures.
- **Storage location:** Describe secure storage servers and access restrictions.
- **Data use:** Define local processing, analysis, and confirm no direct participant contact.
- **Compliance:** State adherence to ethical and legal data protection standards.

**Example (adapted from SDWB):**

Transfer and use in Sweden: Pseudonymized data is transferred to Sunet Drive, a password-protected, MFA-secured service used for research data management at MDU. Data is stored on secure university servers with limited access. The research team conducts data processing, analysis, and model development only—without any direct contact with participants.

All activities comply with institutional ethical standards and relevant data protection regulations.

### 3.5 Key template 5: ethical considerations

#### 3.5.1 Potential risks to research participants

**Purpose:** Identify any risks participation may entail for participants in Sweden.

**Structure:**

- *Type of risk:* Physical, psychological, privacy, or other potential risks.
- *Data handling risks:* Describe how unauthorized access or misuse is prevented.
- *Mitigation measures:* Secure storage, access limitations, encryption, pseudonymization.

**Example (adapted from SDWB):**

The Swedish research involves only secondary analysis of fully pseudonymized motion and activity sensor data collected in Canada. There are no physical, psychological, or privacy risks to participants in Sweden. The main potential risk is data handling; to mitigate this, all data is stored securely on university-administered SunetDrives accessible only to authorized researchers.

#### 3.5.2 Potential benefits for research participants

**Purpose:** Describe benefits for participants, researchers, or society.

**Structure:**

- *Benefits to participants:* Direct or indirect advantages, if any.
- *Societal benefits:* Improvements in care, health outcomes, early detection, reduced caregiver burden.
- *Scientific benefits:* Knowledge generation, methodological advances, publications, professional development.

**Example (adapted from SDWB):**

While no direct benefits accrue to participants, the study benefits older adults in Sweden and beyond. Continuous, non-invasive monitoring enables early detection of health risks, supports timely interventions, promotes independence, reduces caregiver burden, and lowers healthcare costs. Scientific insights advance dementia research, support assisted living services, and facilitate affordable home care.

#### 3.5.3 Risk-benefit evaluation for the project

**Purpose:** Assess balance between potential risks and expected benefits.

**Structure:**

- *Risk assessment:* Evaluate magnitude and likelihood of risks in Sweden.
- *Benefit assessment:* Describe expected short- and long-term benefits.
- *Conclusion:* Summarize whether benefits outweigh risks and justify relevance.

**Example (adapted from SDWB):**

In Sweden, researchers process only pseudonymized movement data with no direct participant contact, eliminating physical or psychological risks. The benefits—developing models for early cognitive decline detection, improving care strategies, supporting caregivers, and fostering scientific knowledge—clearly outweigh any minimal data-handling risks.

#### 3.5.4 Minimization of risks for research participants

**Purpose:** Explain how risks are reduced or eliminated.

**Structure:**

- *Data protection:* Pseudonymization, secure storage, access restrictions.
- *No direct interaction:* Confirm participants are unaffected locally.
- *Ethical compliance:* Adherence to ethical guidelines, laws, and institutional policies.
- *Other measures:* Procedures to prevent misuse, privacy intrusion, training, and long-term data management.

**Example (adapted from SDWB):**

All data is pseudonymized before transfer to Sweden. Researchers have no access to personal identifiers. Data is stored securely on university servers, with access restricted to authorized personnel. No clinical treatments or direct participant interactions occur. The project follows strict ethical and legal standards, minimizing risks while ensuring scientific integrity.

#### 3.5.5 Broader ethical implications

**Purpose:** Consider potential wider ethical issues.

**Structure:**

- *Potential benefits:* Advancement in detection, privacy-preserving methods, support for vulnerable groups.
- *Potential drawbacks:* Misuse risks, bias, or lack of generalizability.
- *Mitigation strategies:* Ethical guidelines, responsible use, data limited to scientific purposes.

**Example (adapted from SDWB):**

The study promotes early dementia detection and privacy-preserving monitoring. Risks include misuse and potential model bias if data is unrepresentative. Strict adherence to ethical guidelines ensures responsible data use, with algorithms applied only for scientific purposes and results interpreted carefully.

### 3.6 Key template 6: reporting of results

#### 3.6.1 Access to data

**Purpose:** Explain how Swedish researchers access study data.

**Structure:**

- *Source of data:* Local or external (e.g., Canadian collaborators).
- *Data transfer and security:* Encryption, secure servers, access controls.
- *Regulatory compliance:* GDPR, PIPEDA, and ethical guidelines.
- *Access restrictions:* Who can access data, under what conditions.

**Example (adapted from SDWB):**

All data is pseudonymized by Canadian collaborators before transfer to Sweden via encrypted channels using MDU’s SunetDrive servers. Access is limited to authorized researchers, ensuring confidentiality. Swedish research involves only processing and analysis; no personal data is accessed. Agreements specify access scope, permitted use, and sharing restrictions. Compliance with GDPR and PIPEDA is maintained.

#### 3.6.2 Responsibility for data processing and reporting

**Purpose:** Identify responsible researchers.

**Structure:**

- *Primary responsibility:* Principal Investigator (PI) or designated team members.
- *Reporting procedures:* Timelines, approvals, and documentation.
- *Publication/dissemination:* How results are communicated.

**Example (adapted from SDWB):**

The PI and authorized research team at MDU handle data analysis and reporting. Results are documented, validated, and prepared for peer-reviewed publications or scientific presentations. Aggregated results only are shared; no identifiable information is included.

#### 3.6.3 Long-term storage and access

**Purpose:** Specify data archiving practices.

**Structure:**

- *Storage duration:* Years and purpose.
- *Security measures:* Encryption, server protections, restricted access.
- *Future access:* Who may access and under what conditions.

**Example (adapted from SDWB):**

Pseudonymized data is stored for at least 10 years on secure SunetDrive servers, accessible only to authorized personnel. Future use requires ethical approval, ensuring long-term integrity and confidentiality.

## 4 Discussion

The preparation of ethics applications for digital health research involves navigating complex regulatory requirements, technical translation challenges, and extensive documentation. While the specific procedures and terminology may vary between countries, these challenges are common across many ethical review systems. Accordingly, the templates presented in this paper, although developed through a Swedish case study, address issues that are widely encountered in international digital health research. Our intention is not to offer guidance limited to the Swedish context, but to present illustrative scaffolds rather than universally generalizable structures. The cross-border nature of the SDWB project also highlights differences in ethical review structures across jurisdictions ethics frameworks.

This paper provides practical, evidence-based templates derived from a successful ethics application for radar-based monitoring of older adults. By sharing these resources, we aim to:

- Reduce the administrative burden on researchers,
- Improve the quality and completeness of applications,
- Accelerate the ethics review process, and
- Support more robust ethical planning in digital health research.

Although the application did not receive formal reviewer comments, feedback from colleagues prior to submission significantly enhanced its clarity, transparency, and rigor. This experience highlights several key considerations for future applicants, particularly for studies involving human participants and international data collection.

Researchers should clearly explain why the studied behavior is considered risky or have potential risks and justify the advantages of the chosen methodological approach. Methodological precision is essential and technologies, data collection, processing, and analysis procedures should be described concretely, including any international data transfers. Ethical and methodological considerations should be illustrated with relevant examples.

Data protection and terminology must remain precise and consistent. Behavioral definitions should be unambiguous, and data handling practices must align with stated policies. Trusted infrastructures, such as Sunet Drive with multi-factor authentication, are recommended for secure storage.

Ethical aspects should be explicitly addressed. Potential benefits, both for research participants and for society, should be clearly described, and consent procedures, particularly for vulnerable populations, must be thoroughly explained. The Swedish Ethical Review Authority places particular emphasis on ensuring the protection of research participants and respect for human dignity (*människovärdet*) (12). Applications should therefore demonstrate how human rights are balanced with the needs of science and society, whether the potential risks are proportionate to the expected benefits, and what measures have been taken to minimize any physical, psychological, or privacy-related risks for participants. If sensitive personal data must be processed, this necessity should be explicitly justified. Special consideration should be given to vulnerable individuals, such as children, persons with cognitive or physical disabilities, refugees, economically disadvantaged groups, or those at risk of discrimination. For such populations, the application should clearly outline safeguards to minimize potential harm and the procedures for obtaining informed consent.

Any prior ethical review conducted abroad should be acknowledged, and responsibilities for risk management and data protection should be clearly defined. Moroever, project planning must ensure that timelines begin only after ethical approval has been granted, with explicit data retention periods and secure disposal procedures specified. Overall, careful attention to conceptual clarity, methodological rigor, data protection, and ethical transparency enhances the quality of ethics applications and provides practical guidance for future researchers. To assist future applicants, the following Box 1 provide a concise overview of the typical ethics application process and timeline.

BOX 1 Ethics Application Timeline.

1. Project design phase: Integrate ethical considerations.
2. 2–3 months before data collection: Begin preparing the application.
3. Submit to Etikprövningsmyndigheten.
4. Review period: Typically 4–8 weeks after receipt of a complete application and fee.
5. Upon approval: Begin data collection.

The Swedish Ethics Review Authority typically issues decisions within 60 days of receiving a complete application and payment. For clinical drug trials involving gene therapy, somatic cell therapy, or genetically modified organisms, the review period may extend to 90 or 180 days depending on required consultations. Approved projects must commence within two years of the decision becoming legally binding, after which the approval expires. Decisions may be approved, conditionally approved, or rejected, and applicants have three weeks to appeal an adverse decision to the Ethics Review Board.

## 5 Limitations

The templates and recommendations we provide are grounded in a small number of cases, specifically one ethics applications (including our own) within the Swedish ethical review system, supported by anecdotal, practice-based observations recorded during preparation and internal peer feedback. As such, they may not capture the full range of disciplinary, institutional, or methodological variations that researchers may encounter in different contexts. Also, they should not be interpreted as predictive of approval outcomes. Ethical review decisions depend on project-specific risk profiles, disciplinary norms, and evolving regulatory interpretations. The materials provided here are intended to support ethical clarity and reflexive reasoning rather than to guarantee procedural success.

The international perspective illustrated in this paper is based on a single cross-border collaboration between Sweden (as a centralized ethics review environment) and one non-EU country (Canada as a decentralized institutional Research Ethics Boards). Although many of the principles discussed such as transparency, data governance, and cross-border data protection are broadly relevant, the regulatory conditions, documentation requirements, and support structures differ across jurisdictions. These variations underscore the importance of explicit articulation of responsibilities, data governance mechanisms, and cross-border compliance in international digital health research. The guidance and templates provided in this study should therefore be viewed as field-tested and illustrative scaffolding, rather than generalizable empirical findings, that requires tailoring to project–specific risks, participant populations, and data flows; it does not substitute for formal institutional advice or legal review. Future work should expand the evidence base by systematically comparing multiple applications across institutions and jurisdictions and by prospectively evaluating whether use of these templates measurably shortens review times or reduces clarification requests.

## 6 Conclusions

This paper addresses a critical gap in ethics application resources by providing practical, field-tested templates for digital health research in Sweden. Drawing from a successfully approved ethics application for radar-based monitoring of older adults, we present reusable structures covering the most challenging aspects of Swedish ethics applications: articulating research in accessible language, describing complex technologies, justifying data practices, addressing vulnerable populations, and ensuring GDPR compliance in cross-border research.

In the Swedish research context, structured support for ethical competence is well established in fields such as medical sciences, caring sciences, and the social sciences. Doctoral education in these areas often includes mandatory coursework in research ethics, and universities typically provide advisory support through Ethics Councils or similar bodies. However, comparable formalized training and advisory structures may be less consistently available in technical disciplines, underscoring the potential value of complementary functions to support ethical reflection in technology-oriented research.

Our experience demonstrates that successful applications require ethics review to begin during project design rather than afterward, allowing ethical considerations to shape methodology from the outset. Structured templates reduce oversight while ensuring comprehensive coverage, and plain-language explanations alongside technical details facilitate reviewer understanding. Concrete demonstrations of GDPR compliance with specific storage, security, and access details, prove more effective than general assurances. The interdisciplinarity of the input of healthcare, legal and technical experts strengthens both ethical reasoning and practical feasibility. The first-submission approval without reviewer comments validates this approach’s alignment with the Swedish Ethics Review Authority’s expectations.

These templates serve as practical scaffolds that support, but cannot replace, careful ethical reasoning and institutional guidance. They are based on successful Swedish ethics application involving international collaboration, where data collection was conducted abroad and data processing occurred in Sweden. It primarily reflects observational, sensor-based monitoring studies and may require adaptation for interventional research or other technological contexts. While optimized for the Swedish regulatory environment, the principles may be transferable to other EU countries. Users should also consider variations in ethical review practices and institutional requirements that may not be fully captured by the templates.

In fact, each project requires thoughtful analysis of its unique ethical dimensions beyond template completion. We invite researchers to share experiences using these materials, suggest improvements, and collaborate on developing similar resources for other jurisdictions. We plan to track adoption, gather feedback, update templates as regulations evolve, particularly with EU AI Act implementation, and develop companion guidance for consent forms and other ethics documents.

By reducing administrative burden and improving application quality, these templates aim to support more efficient ethics review processes and ultimately contribute to more ethically robust digital health research.

## Footnotes

^1^ https://etikprovningsmyndigheten.se/

^2^ https://www.es.mdu.se/projects/647-Secure_Detection_of_Wandering_Behavior__Indoor_Monitoring_Enhanced_by_Deep_Learning_

^3^ https://www.chalmers.se/aktuellt/nyheter/e2-alzheimers-och-fallolyckor-kan-forutspas-med-hjalp-av-radar/
